# Current Progress and Future Prospect of Wheat Genetics Research towards an Enhanced Nitrogen Use Efficiency

**DOI:** 10.3390/plants12091753

**Published:** 2023-04-25

**Authors:** Yun Zhao, Shahidul Islam, Zaid Alhabbar, Jingjuan Zhang, Graham O’Hara, Masood Anwar, Wujun Ma

**Affiliations:** 1 Food Futures Institute & College of Science, Health, Engineering and Education, Murdoch University, Perth 6150, Australia; zhaoy47249@126.com (Y.Z.); s.islam@murdoch.edu.au (S.I.); j.zhang@murdoch.edu.au (J.Z.); g.ohara@murdoch.edu.au (G.O.); masoodanwar84@gmail.com (M.A.); 2 Institute of Cereal and Oil Crops, Hebei Academy of Agriculture and Forestry Sciences, Laboratory of Crop Genetics and Breeding of Hebei, Shijiazhuang 050035, China; 3 Department of Plant Sciences, North Dakota State University, Fargo, ND 58108, USA; 4 Department of Field Crops, College of Agriculture and Forestry, University of Mosul, Mosul 41002, Iraq; alhabarzaid@yahoo.com; 5 College of Agronomy, Qingdao Agriculture University, Qingdao 266109, China

**Keywords:** wheat, nitrogen use efficiency, grain yield, grain protein content, QTLs, molecular markers

## Abstract

To improve the yield and quality of wheat is of great importance for food security worldwide. One of the most effective and significant approaches to achieve this goal is to enhance the nitrogen use efficiency (NUE) in wheat. In this review, a comprehensive understanding of the factors involved in the process of the wheat nitrogen uptake, assimilation and remobilization of nitrogen in wheat were introduced. An appropriate definition of NUE is vital prior to its precise evaluation for the following gene identification and breeding process. Apart from grain yield (GY) and grain protein content (GPC), the commonly recognized major indicators of NUE, grain protein deviation (GPD) could also be considered as a potential trait for NUE evaluation. As a complex quantitative trait, NUE is affected by transporter proteins, kinases, transcription factors (TFs) and micro RNAs (miRNAs), which participate in the nitrogen uptake process, as well as key enzymes, circadian regulators, cross-talks between carbon metabolism, which are associated with nitrogen assimilation and remobilization. A series of quantitative genetic loci (QTLs) and linking markers were compiled in the hope to help discover more efficient and useful genetic resources for breeding program. For future NUE improvement, an exploration for other criteria during selection process that incorporates morphological, physiological and biochemical traits is needed. Applying new technologies from phenomics will allow high-throughput NUE phenotyping and accelerate the breeding process. A combination of multi-omics techniques and the previously verified QTLs and molecular markers will facilitate the NUE QTL-mapping and novel gene identification.

## 1. Introduction

Wheat is one of the most important crops in the world and an essential source of starch and protein for human beings. It was the first cereal known to be domesticated around ten thousand years ago and has been a staple food for people living in Europe, Western Asia and North Africa ever since [1]. The global wheat production in 2020 was 761 million metric tons (MMT) harvested from 219 million ha, with an average yield of 3.47 tons ha^−1^ (FAO, 2021). Since there is little scope of increasing the land area, a major yield increase is expected to meet the increased global requirement due to a fast population growth. An increase in nitrogen (N) fertilizer application is one way for the increment of wheat grain yield (GY). The global agricultural use of N fertilizer was 108 MMT in 2019, and the fertilizer use is projected to increase to 236 MMT by 2050 to meet the global demands [2]. However, a sustainable N fertilization regime is required in order to reduce or even eliminate environmental damage due to N pollution [3,4].

Nitrogen fertilizers account for a very large part of energy use in crop production since most plants cannot directly use nitrogen gas which accounts for 78% of the atmosphere [5], and wheat is the main crop receiving N fertilizers, ahead of maize and rice [6]. The N availability from soil and the applied N fertilizers determines the plant rate and the final yield and protein content in wheat production. Studies by Justes et al. [7] showed that the reduction in grain number and grain weight is linearly related to N deficiency at anthesis. A reduction in the rate of leaf production, leaf number, number of nodes, number of tillers have also been reported in wheat in N limited condition [8]. However, crops tend to have a low NUE (use only 40–50% of the applied N), the rest of the applied N fertilizer is unavailable to the plant and will cause N pollution [9,10]. In wheat, the recovery of applied N is very low, with only 33% ending up in the grain; the major reason for this is the poor N uptake efficiency of cereals [11].

Nitrogen is not only an essential element for wheat growth, but also one of the major determinants for wheat yield and protein quantity. Therefore, it is vital to improve NUE in the pursuit of higher wheat yield and better protein quality. A number of fertilizer application strategies have been implemented to achieve this goal, including using nitrate soil tests, improved timing of N application at appropriate rates, plant monitoring, diversifying crop rotations, using cover crops, reducing tillage, optimizing N application techniques, and using nitrification inhibitors [12,13]. To avoid N leaching, the US Code practice for N fertilization encourages use of nutrient management technologies such as incorporation or injection of stabilizers, slow-release fertilizers, organic manures, constructed wetland, conservation stream buffer (2013, Code practice for N fertilization, United States). However, only a short-term improvement of NUE can be achieved through the above agronomic practices, which are also not economically efficient. While in the long run, the inherent ability of the crop to uptake more available N and use it more efficiently for high grain and protein yield needs to be tackled genetically [14]. Wheat NUE is an important quantitative trait that is highly complex. It is easily influenced by the environment and its controlling network is still not clear [4]. Nevertheless, through genetic selection, modern cultivars have gained an enhanced NUE and out-yielded the older cultivars. A study of 183 cultivars released after 1960 demonstrated a trend of an increase in grain N yield, GY, N harvest index (NHI), N uptake efficiency (NUpE), N utilization efficiency (NUtE) and post-anthesis N uptake [15]. The study by Cormier et al. [16] also indicated the same trend towards an improved NUE, NUtE and NHI over the last 25 years using a set of elite European winter wheat germplasm. These results suggested the existence of a great potential for increasing NUE through the genetic approach.

Focusing on bread wheat crops, the objectives of this review are to (1) summarize the relationship of NUE with GY and GPC; (2) discuss the factors that play major roles in NUE; (3) collect the published QTLs and markers linked with NUE and GPC, and (4) provide some perspectives for future research directions.

## 2. Definitions of NUE and Important Traits Associated with NUE

An appropriate determination of NUE in crop plants is crucial to evaluate the fate of applied N fertilizer and its role in improving crop yields [11,17]. Considering various aspects of N use, which include the uptake, assimilation and translocation of N in plants, several definitions of NUE have been discussed and reported [17,18,19,20,21]. NUE can be described depending on whether the focus is on grains only or on the total biomass. The most commonly used definition is reported by Moll et al. [22] who defined NUE as the yield of grain per unit of available N in the soil (NUE = Gw/Ns. Gw, grain weight; Ns, nitrogen supply). To measure the use efficiency of applied N fertilizer, definitions of NUEs have been further classified as agronomic efficiency (AE), defined as the economic production obtained per unit N applied (AE = (Gw_Fert_-Gw_Con_)/N_F_. N_F_, N fertilizer applied; Gw_Fert_, grain weight with fertilizer; Gw_Con_, grain weight of unfertilized control), and physiological efficiency (PE), defined as the biological yield (grain plus straw) obtained per unit of N uptake by both grain and straw (PE = (Gw_Fert_-Gw_Con_)/(N_Fert_-N_Con_). Gw_Fert_, grain weight with fertilizer; Gw_Con_, grain weight of unfertilized control; N_Fert_, plant N with fertilizer; N_Con_, plant N of unfertilized control). Both definitions address only the importance of grain weight in the calculations but completely ignore the trait grain protein which is the only determinant of grain N content. Based on those calculations, high NUE does not indicate the efficient transfer of N to the grains, rather GY is principally contributed by the efficient transfer of carbohydrate to the grain.

Considering the N (protein) content of the grain another way of measuring NUE has been reported extensively by calculating NHI (NHI = Gw * N%/Nt. Gw, grain weight; N%, grain N content; Nt, total N in plant). NHI is the translocation efficiency of acquired N for grain protein accumulation [17,23], and clearly, a greater NHI will reflect lower losses of applied fertilizer and an efficient N utilization/translocation. This parameter is of particular importance since it emphasize both NUE and GPC, the latter determines wheat price [24]. GY is heavily contributed by carbohydrate (starch) accumulation in the grain. However, the high proportion of starch in the grain has the effect of diluting grain protein/N content. Hence, there is an inverse relationship of yield and grain N. Thus, it is extremely difficult to improve GY and GPC simultaneously which only can be achieved by a large N input, that ultimately results in a low NUE overall. Nevertheless, genetic sources of a deviation from this relationship have been reported which is commonly known as grain protein deviation (GPD) [25]. A positive GPD refers the case of a simultaneously high starch and high protein content, which therefore leads to a high NUE. It is worth mentioning that GPD is under genetic regulation with a considerable variation between genotypes [26,27], and QTLs associated with increased GPD have been reported [28].

Overall, NUE can be divided into two processes: uptake efficiency (NUpE), the ability of the plant to uptake N from the soil normally present as nitrate and ammonium ions; and utilization efficiency (NUtE), the ability of the remobilizing N accumulated in sink organs to the grain [2]. NUpE is the ability to take up and store N from the soil and will depend on root architecture, longevity and functioning. Studies have indicated that N uptake directly linked to dry matter accumulation. NUtE is the efficiency of carbon fixation for the N taken up and includes processes involving photosynthesis, canopy formation, activity and longevity, as well as nutrient remobilization from all tissues to grain during seed filling. Optimum NUtE is ideal for cereal crops grown in low fertility where plant-available N is limited. In wheat, accumulation and redistribution of N are crucial processes in determining yield quantity and quality. Several studies report that the contribution of NUtE to overall NUE is not well understood and may not be as significant as NUpE in wheat, barley, oats and maize [29,30,31]. High levels of N led to the decrease in N mobilization as increased post-anthesis uptake makes N remobilization less necessary; while low N increased mobilization. Heat and water stress also increase NUtE as plants try to make great use of N accumulated at anthesis [5]. Straw N concentration was reported to have a significant positive (*p* < 0.01) correlation with N translocation and translocation efficiency. Straw N concentration adequately represents NUtE for synthesis of grain protein. It is a simple parameter, simpler than NHI, since less work needs to be done for straw N concentration determination than for NHI. This trait could be recommended as an alternative indicator in wheat NUtE evaluation [32].

GPD is linked to anthesis date and post-anthesis N-uptake or to grain-specific processes reflected by intrinsic grain gene expression profiles. GPD may be affected by partitioning, as a large fraction of grain N comes from remobilization from vegetative tissues and is quantified the NHI [33]. Sustainable crop production requires inputs matched to outputs and hence principal gains are to be achieved by minimizing losses from the system (run-off, leaching and volatilization) by optimizing application and uptake, or by improving NUE and NHI. Improving NUE without increasing NHI will lead to a lower GPC which ultimately will have a negative impact on price.

## 3. Genetics of the Negative Correlation between GY and GPC

While the genetic basis behind biotic stress resistance of wheat is relatively well understood for the prevalent diseases, the genetic basis of cultivar level differences in yield potential and protein deposition is not clearly understood [34], which might be due to quantitative genetic regulation of these traits by the multiple genes. Both GY and GPC are crucial traits associated with NUE, which can be reflected by their importance in wheat production as well as in the calculation of NUE. However, a negative correlation between GY and GPC has been constantly reported in previous studies [35,36,37]. This negative relationship might be related to the presence of genes having pleiotropic effects on both GY and GPC through a dilution effect by carbohydrates, such as genes affecting the duration of leaf senescence after flowering [38,39]. It could also be explained by the competition of GY and GPC for energy resources and the interactions between C and N assimilation processes [26].

Attempts have been made to break this negative correlation between GY and GPC. GPD, as a trait that emphasizes both GY and GPC and reflects their interaction, should be considered as a valuable indicator for a balanced NUE [33]. An analysis of 27 genotypes indicated that GPD was significantly correlated with post-anthesis N uptake independently of anthesis date and total N at anthesis [26]. Selection for varieties with high post-anthesis NUpE can be a good option of improving GPC without reducing GY. It could also be supported by late N fertilizer application around the heading stage achieved similar results. This suggested the presence of genes having independent effects on GY and GPC, indicating that increasing NUtE or post-anthesis N uptake would allow increases in GPC without decreasing GY [40]. A study of effects of 75 parameters on GY and GPC under high and low N using a in silico system suggested that almost all traits had opposite effects on GY and GPC expect the leaf and stem N storage capacity, which appeared as good candidate traits to alleviate the negative correlation between them [41].

Cultivars with elevated GPC of wheat are more efficient at N remobilization from vegetative tissue to grains [42] and GPC is also thought to be influenced by the amount of N taken up after anthesis [43]. Because the majority of grain N originates from remobilization [44], mechanisms to enhance reserve N accumulation in the canopy and efficiency of N remobilization should be addressed in the genetic improvement of GPC [45,46].

Even though there exist possibilities to improve GPC and GY simultaneously [36], it was reported that chromosomes 2A, 2D, 3B, 7B and 7D harbored pleiotropic QTLs for GPC and GY with antagonistic effects. Nonetheless, QTL regions determining GPC independently of GY across experiments were identified on chromosomes 3A and 5D which could help breeders to remove the negative GPC-GY relationship in a desirable direction [40]. It is still very challenging since unless a major gene is identified, the genetic effects of minor genes can usually be masked due to the large genotype by environment (G×E) interactions [26].

## 4. NUE Variability Influenced by N Level

A study by Van Ginkel et al. [47] indicated that, under high N input, high NUpE is a desirable trait describing NUE whereas under low input system the development of cultivars with high NUtE is considered more desirable. It has been widely reported that crop species and genotypes within species differ significantly in NUE [17,20,48,49]. Effects of genotypes on NUE in wheat can be reflected by their contributions to the expression of either NUpE or NUtE under different N rates [50,51,52]. These differences on genetic variability were further confirmed by detection of specific QTLs for a given rate of fertilization [53,54,55,56,57]. These results suggest that several sets of genes are differentially expressed according to the amount of N provided to the plant. Therefore, different N rates should be considered in QTL mapping studies associated with NUE in wheat.

Most of the varieties released after the ‘Green Revolution’ are better adapted to optimum fertilizer condition and can only achieve ideal yield under relatively high N input since those cultivar selections were conducted under optimum condition [20]. To achieve a ‘Second Green Revolution’, wheat varieties must have the ability to uptake N fertilizer efficiently and produce reasonable yield in marginal conditions.

In recent years, marker-assisted selection (MAS) have been widely and effectively used in modern wheat breeding program ever since the functional polymorphism were identified of the diagnostic molecular markers [58,59], those markers include homoeoallelic series at phenology genes including *Ppd*, *Vrn*, *Eps*; genes governing plant height such as *Rht* genes; disease-resistant genes, e.g., leaf rust *Lr* genes and powdery mildew *Pm* genes, etc. Diagnostic assays have also been developed and used for alien introgression segments including 1BL.1RS rye translocation and *Agropyron* translocation [60]. A vital source of molecular markers is a QTL mapping study on various traits of great significance. QTL mapping of NUE and its related traits using breeding materials showing variations in NUE [53,54,55,56,57,61] have produced a series of useful and robust markers towards the molecular breeding for wheat varieties with higher NUE.

## 5. Genetic Regulation of Nitrogen Uptake

**Figure 1:**
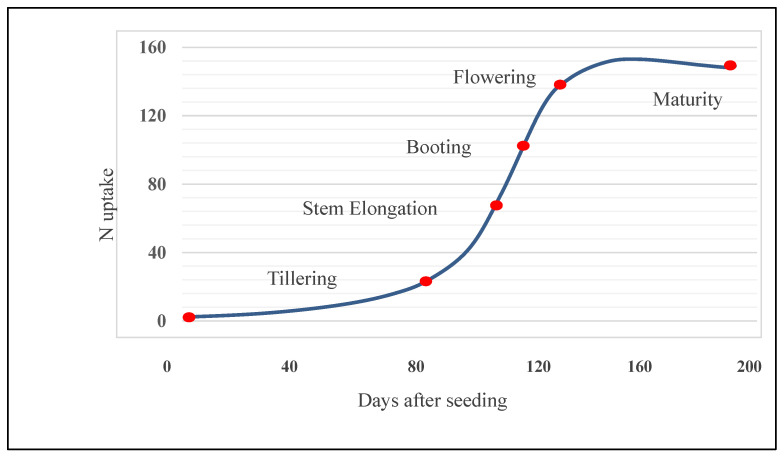
Nitrogen uptake at different growth stages of wheat [65].

Nitrate is readily transported in both xylem and phloem but very little NH_4_^+^ is transported through the plant because of its toxicity. The other main forms of N transported are amino acids and amides [11]. Although the physiological and genetic basis of variation in N uptake is not fully understood, root morphology and stay-green properties are thought to be crucial [62,63]. Improvements in N fertilizer application can help to improve N uptake. Post-anthesis foliar N fertilization was reported an effective approach [64]. N uptake from foliar spraying was found to have the advantage of being less dependent on soil moisture and maybe effective when root uptake is impaired in dry soils. Lower quantities of N are required for foliar spraying, thus minimizing phytotoxicity and leading to variety-dependent up-regulation of essential low molecular weight glutenin subunits (LMW-GS) and gliadins. The maximum N uptake occurred in the pre-anthesis phase as the root system expanded and leaves and stems developed, while that declined during plant maturation and the grain filling phase. In wheat, the period of the highest N uptake matched with the time of rapid biomass accumulation, which ranged between tillering and flowering stages (Figure 1).

**Figure 2:**
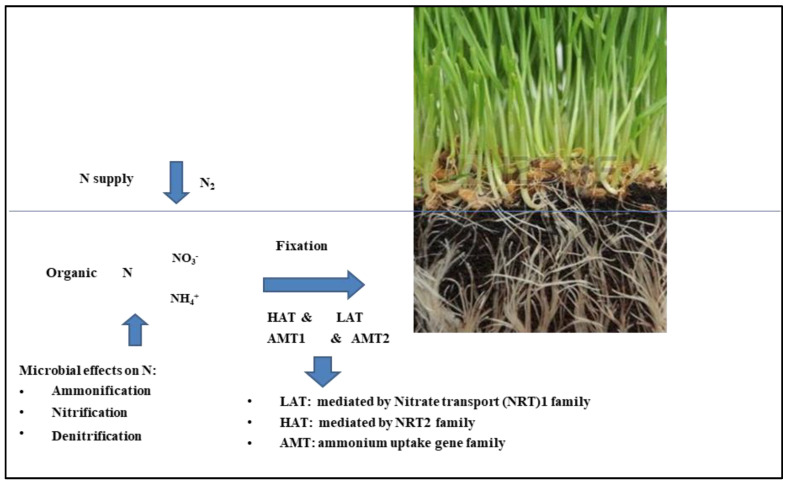
Nitrogen uptake through LATS and HATS.

In recent years, more attention has been attracted to understanding and utilizing the genes involved in N uptake. High and low affinity transport systems, namely high-affinity ammonium transport system (HATS) and low-affinity ammonium transport system (LATS) play major roles in the nitrate uptake by roots. These transporters belong to the NRT2 and NRT1/NPF families and they are both represented by multiple genes [11]. LATS are a group of peptide transporter proteins and are encoded by *NRT1* gene, while HATS are a group of nitrate-nitrite porter proteins encoded by *NRT2* gene. Genes control the two transport systems for ammonium uptake are the gene families *AMT1* and *AMT2*. Uptake of NO3^−^ and NH4^+^ is downregulated by the high plant N status and downstream assimilates such as amino acids [66] (Figure 2).

In plants, N sensing is a rather complicated process (reviewed by Reddy et al. [67]). Ca^2+^ dependent protein kinases (CDPKs) and CBL-interacting protein kinases (CBL/CIPKs) were reported to play important roles in the process [68]. Calcium binding CBL can activate certain CIPKs, which regulate the phosphorylation of NRT1.1 under different nitrate rates, thus mediating the nitrate sensing of the plant plasma membrane. A case in point is the interaction of calcium binding CBL9 protein and CIPK23 kinase under low nitrate concentration to turn on the high affinity mode of nitrate sensing. As for ammonium sensing, different CDPKs and CIPKs can be differentially induced in response to different ammonium levels. In addition to the above two types of transporters, chloride channels (CLCs), slow anion channel-associated 1 homolog 3 (SLAC1/SLAH), and aluminum-activated malate transporters (ALMT) are also involved in nitrate uptake process [69,70].

A number of transcription factors (TF) that regulate N transporters also play important roles in N uptake, such as MADS-box TF ANR1, LOB Domain-Containing proteins (LBD37/38/39), Teosinte branched 1/Cycloidea/Proliferatingcell Factor 1–20 (TCP20), NIN-like proteins (NLP6, NLP7), Nitrate regulatory gene 2 (NRG2), Nuclear factor Y (NF-YA), High nitrogen insensitive 9 (HNI9), Hypersensitivity to Low Pi-Elicited Primary Root Shortening 1 (HRS1), to name a few (reviewed by Plett et al. [71]). It was also reported that some of the transcription factors or genes are induced by nitrogen starvation condition, such as basic leucine zipper (bZIP) transcription factor gene, AtTGA4, cytokinin synthesis gene isopentenyltransferase (IPT) [72]. Manipulating those genes through genetic approach can potentially improve nitrogen uptake.

Transcriptional Micro RNAs (miRNAs) have emerged in recent years as another mode of governing gene expression in plants. Studies have now revealed that significant differences in miRNA accumulation are observed in response to nitrate availability [4], especially under low NO_3_^−^ conditions. The expression of miRNAs, including miR528a/b, miR528a*/b*, miR169i/j/k, miR169i*/j*/k*, miR167, miR169 and miR393, have been studied in maize and Arabidopsis and shown to be involved in regulating nitrate responses (reviewed by Plett et al. [71]). Their roles in regulating nitrogen sensing in wheat deserve more exploration.

## 6. Genetic Basis of Nitrogen Assimilation and Remobilization

**Figure 3:**
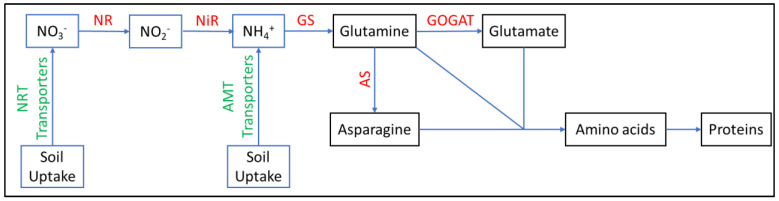
Nitrogen assimilation pathway.

The majority of nitrogen in the wheat grain (51–92%) is derived from remobilization of nitrogen from the vegetative parts of the plant [43]. Nitrogen availability, environment conditions and genotype greatly influence the remobilization efficiency [73]. The storage capacity of nitrogen in the canopy, time of onset of leaf senescence, capacity of sink organs were all found to influence nitrogen remobilization [8,52,74]. The nitrogen stored in the vegetative parts involves a series of transportation and assimilation. After being uptaken by roots, nitrate assimilation occurs first through nitrite reductase (NR) to NO_2_^−^ and then via nitrate reductase (NiR) to NH_4_^+^, ammonium is then assimilated into organic nitrogen via glutamine synthetase and glutamate synthase (GS/GOGAT) [11] (Figure 3). Based on subcellular localization, two categories of plant GS exist, including GS1 and GS2. The former is localized in the cytoplasm and the latter is localized in the plastid. In wheat, four types of GS genes were reported, including GS1, GS2, GSr, and GSe that are located on homologous groups 6, 2, 4 and 4, respectively [73]. Both GSr and GSe are localized in the cytoplasm [75]. There are also two isoforms of GOGAT, including the ferredoxin dependent (Fd-GOGAT) and the NADH dependent (NADH-GOGAT). Fd-GOGAT plays an important role in leaf photo-respiratory ammonium assimilation and preferentially work with plastidic GS2. Whereas NADH-GOGAT combines with cytosolic GS1 to assimilate NH_4_^+^ produced by nitrogen-fixing bacteria [19]. The *Fd-GOGAT-A* gene was found co-localized with a major QTL for GPC and was located on the short arm of chromosome 2A [28].

Despite that the genes that control key enzymes are directly involved in nitrogen metabolism, other regulatory genes are also considered important in nitrogen assimilation [39,76]. Circadian clock master regulator, circadian clock-associated 1 (CCA1) was reported to control the expression of genes involved in N assimilation and established a link between nitrogen metabolism and circadian clock [77]. miR5640 was confirmed to target phosphoenolpyruvate carboxylase (PEPC) which is vital in maintaining C/N balance [77]. Other transcription factors such as NLP7, Dof1, GATA were also found to play important roles in regulating nitrogen assimilation process [72,78]. An approach using methionine sulphoximine to inhibit glutamine synthetase and prevent ammonium assimilation identified many genes responding to downstream reduced nitrogen-containing compounds rather than nitrate as previously assumed [79]. This study highlighted the involvement of the circadian clock in controlling N assimilation as well as a possible influence of N nutrition on clock functioning. This regulatory interaction is an adaptive response to coordinate cellular metabolism during changing diurnal conditions and is another factor that must be taken into account when considering NUE in wheat. A transcriptome analysis of senescence in the flag leaf of wheat over a time course following ear emergence identified 140 up-regulated genes with informative annotations, including genes involved in macromolecule degradation and nutrient remobilization, as well as NAC-domain and WRKY transcription factors [80]. Processes in stems also contribute to grain filling: stem water-soluble carbohydrates can remobilize to grain which remobilization may be enhanced by nitrogen limitation. Comparing with the high nitrogen supplied plants, genes involved in fructan biosynthesis are up-regulated in nitrogen limited plant stem but are then down-regulated upon sucrose feeding of individual culms [81]. Differential expression of proteins was observed in low-N-sensitive and low-N-tolerant maize genotypes in response to various N treatments [82]. This suggests that protein profiling could be a way to select well-performing genotypes for reduced N fertilization.

## 7. Genetic Approaches to Improve Wheat NUE

Genes involved in nitrogen metabolism have been manipulated through transgenic approaches in multiple studies reviewed by [11,63,83], although reports on wheat are still limited. Transporter genes including nitrate transporters, ammonia transporters, amino acid transporters; key enzymes including GS, asparagine synthetase (ASNS), GOGAT, asparaginase, aspartate aminotransferase (AspAT), alanine aminotransferase (AlaAT), Glutamate dehydrogenase (GDH); TFs including NAC, WRKY, NF-YA and miRNAs can all be used for lifting NUE [4,19]. The manipulation of expressional status of a number of NUE-related known enzyme genes has been reported to increase the NUE. For example, over-expression of a rice *NRT2* gene was reported to improved nitrate-uptake capacity, C metabolism and GY [84]. Over-expression of a tobacco nitrate reductase gene (*NtNR)* in two commercial winter wheat cultivars, ND146 and JM6358, resulted in a remarkable enhancement of foliar nitrate reductase activity and insignificantly augmented GPC and thousand kernel weight (TKW) in the majority of the T1 offspring analyzed [85]. Likewise, over-expressing *TaGS2* in wheat significantly increased spike numbers per plant, TKW and GY [86]. Crop plants over-expressing *AlaAT* using a *PBpr1* promoter, which is a promoter for genes encoding methyl-melonate semialdehyde dehydrogenase (*MMSDH*) have enhanced NUE [87], which is rather surprising as AlaAT was previously not thought of as a key component of nitrogen metabolism. Over-expression of two glutamine synthetase genes *Gln1–3* and *Gln1–4* in maize improved yield and enhanced NUE [88].

AspAT plays important roles in providing precursors for biosynthesis of amino acids of the aspartate family and major nitrogen transport molecules such as asparagine and ureides. The gene encoding AspAT was significantly upregulated at high concentrations of ammonium, indicating an enhanced process for amino acids biosynthesis while another enzyme histidine kinase 1 was upregulated at a low ammonium concentration [68], suggesting their roles in nitrogen metabolism and potential in NUE enhancement. Altering the expression of several TFs has also been demonstrated to improve NUEs in wheat. *TaNAC2–5A* is one of those TFs, which plays a role in nitrogen metabolism pathways, transgenic plants of over-expressed *TaNAC2–5A* displayed enhanced root growth, nitrate influx rate, and GY [89]. Similarly, the over-expression of *TaNAC-S*, a member of the NAC transcription factor family, resulted in delayed leaf senescence and increased GPC, while the crop biomass and GY remained unaffected [85]. The Nuclear Factor Y (NF-Y) transcription factors are also recognized as important regulators of many plant developmental and physiological processes. TaNFYA-B, a low-nitrogen- and low-phosphorus-inducible NFYA transcription factor, was over-expressed in wheat that led to a significant increase in nitrogen and phosphorus uptake and GY in a field experiment [90]. Transcription factor, 14–3-3 proteins have been involved in the regulation of several cellular processes [91]. They are found to regulate nitrate reductase and glutamine synthetase in an N-dependent manner.

Since photosynthesis is a major yield contributing factor, improvement of NUE by altering photosynthesis related genes have also been tested in wheat (Wang et al., 2019). It is worth noting that carbon assimilation in wheat (C3 plant) can be improved by ectopically expressing individual or multiple genes (*PEPC*, *PPDK*, etc.) of the photosynthetic pathways of C4 plants. Transgenic wheat plants over-expressing genes encoding phosphoenolpyruvate carboxylase (PEPC) and pyruvate orthophosphate dikinase (PPDK) simultaneously, which showed a positive synergistic effect on wheat photosynthetic characteristics and yield [92]. *TaWRKY51* over-expression lines showed a higher number of lateral roots than the wild type, with the potential of improved NUpE [93]. The occurrence of miRNAs and long noncoding RNAs (lncRNAs) has been investigated in plants grown under different levels of N supply [94]. Genetic manipulation of plant hormone cytokinins may influence several physiological processes, some stress tolerances, root formation and crop yield. Phenotyping of the transgenic lines of silenced *HvCKX1* gene revealed a reduced root growth but more tillers and grains than the azygous wild-type controls, which resulted in a total yield increase of 15% [95]. Proteins participating in intracellular sensing of carbon and nitrogen levels as well as energy and redox maintaining and signal transduction were also found involved in the regulation of nitrogen metabolism. Those proteins include PII protein, glutamate receptors, NRT2.1, SNF1/AMP-dependent kinase, the ubiquitin ligases ATL31 and ATL6, trehalose-6-phosphatease, etc. [67].

It is worth noting that single gene transgenics have not always led to significant changes in NUE [11,83], which is expected as NUE is a quantitative trait that involves a coordinated expression of a series of genes related to nitrogen metabolism [13,39,96]. Moreover, genes discovered and verified to be related with NUE in other crop species might not be effective in wheat. Therefore, a full understanding and simultaneously manipulation of different genes involved in nitrogen metabolism are needed to efficiently improve wheat NUE.

## 8. QTLs and Markers Associated with GPC

GPC is an important trait directly associated with NUE, but it is often overlooked in breeding compared with GY. Of a mature wheat grain, the starch content accounts for 55–75% of the total grain dry weight and proteins possess another 10–20% [97]. Nitrogen and sulphur availability as well as genotype are the most critical factors determining GPC [76,98]. The genetic heritability accounts for about one third of GPC variation [99], suggesting it is worthwhile to explore the genetic potential for GPC improvement. Although a great number of genetic studies have been carried out, there is a lack of proper reviews and summaries of QTL mapping and marker development for GPC.

Grain protein not only determines the nutritional quality and baking properties of wheat, but it is also an important source of nutrition to support human health [100,101]. Wheat classification factors, kernel hardness and GPC, can define the functionality of the grain, the type of milling process and the nature of the milled product [102]. However, many wheat breeding programs have emphasized GY, resulted in a lower GPC in modern cultivars than in historical cultivars [42]. A balanced GY and GPC in wheat is essential in maintaining an optimal NUE, and more efforts should be placed on deciphering the genetic mechanism behind wheat GPC. So far, a number of studies have been carried out to identify major genes in wheat associated with GPC through QTL mapping approach (Table 1), and various genetic regions were found to have effects on wheat GPC, while only few have been used in wheat breeding.

It was reported that QTLs for GPC were co-located with *Ppd-B1* and *Ppd-D1* gene region [119,144], these loci were also reported to have effects on plant development and kernel size [157]. Results from these experiments suggested that early flowering conferred by photoperiod insensitivity allowed for longer grain filling, which resulted in larger grain with lower GPC. A significant negative correlation between TKW and GPC was also reported in another study [145]. A few phenology gene loci on 3B and 5A were found to be associated with GPC and were considered useful in improvement of both GY and GPC through genetic gain, although phenology genes such as *Vrn*, *Ppd* and *Eps* mainly determine the wheat pre-anthesis development [158]. Regulation of seed number per spike related genes including FRIZZY PANICLE [159], *bht-A1* [160], TEOSINTE BRANCHED1 (*TB1*) [161], were found to have the potential to improve yield. Similarly, regulation of floret numbers per spike related genes including Q [162]. APETALA 2-like gene [163], miR172 [164] and Grain Number Increase 1 (*GNI1*) [165] could also lead to yield increase. Furthermore, the mechanisms of Q locus regulating various aspects of the spike development such as spike compactness, threshability, and spikelet determinacy have been recently revealed [162,164,166] which can be further characterized for wheat NUE improvement. Moreover, better understandings on spike and spikelet development can also be obtained by comparisons between wheat and other Triticeae species or other crops such as barley and maize [167].

Cristobal Uauy et al. [168] cloned from a *Triticum turgidum*/*ssp. durum* population a NAC transcription factor, called *NAM-B1*, which is the causal gene underlying *Gpc-B1* QTL on chromosome 6BS. This gene was shown to accelerate canopy senescence during grain filling and to be responsible for a higher nitrogen remobilization and a better partitioning of nitrogen to the grain, it has been reported to simultaneously improve both GY and GPC [74,169]. An association mapping study of 158 barley accessions also confirmed the remarkable positive function of *NAM* gene, as major QTLs associated with GPC were located on 6H and 2H that were closely linked with *HvNAM1* and *HvNAM2* gene, respectively [170].

With the reports on GPC QTLs collected, it is worthwhile to compare the marker information related to those QTLs from the literature especially for those repeatedly reported ones, which will be helpful in breeding programs for GPC improvement. Table 1 listed the markers published previously for GPC. The table shows that some genetic regions were reported in more than three publications, including *Xbarc15-Xgwm558-gwm614* on 2A, *Xwmc245-Xgwm271-barc0013* on 2B, *Xwmc3-Xwmc56-wmc418* on 3B, *Xgwm368-Xwmc617-Rht-B1* on 4B, *Xcfd193-Xcfd71-wmc457* on 4D, *Xgwm540-Xgwm499-BE495277_339* and *Xbarc234.1-Xfcp273-XwPt9006* on 5B, *Xwmc215-Xcfd29-Xbarc320* on 5D, *Xcfd80.2-Xbarc1055-Xbarc37* on 6A, *Xgwm133-Xbarc24-Xgwm219* on 6B, *Xgwm573-Xwmc9-ksuH9* and *wmc017-NW1257-Xcfa2174.1* on 7A, *Vrn-D3-wPt-3727-Xgwm295* and *Xgwm111* on 7D. It can also be concluded from this table that QTLs for GPC often show pleiotropic effects for TKW owning to their significant correlation. Repeatedly published markers were found on almost all chromosomes except for 1D and 3D. Marker *Xcfd18* on 5D was also reported to be associated with *GS2* gene [171]. Genes such as *Glu-B1*, *Ppd-B1*, *Ppd-D1*, *FdGOGAT-B*, *Ha*, *Vrn-D1*, *Vrn-D3* were found to be co-localized with GPC QTLs. A strategic integration of those genes in future GPC improvement should be taken into consideration. On the other hand, avoidance of segregation of those genes in future GPC QTL-mapping studies should also be noted in order to identify new QTLs.

## 9. Future Directions

Generally, GY is the most important criterion for selection in conventional wheat breeding for NUE. However, the yield is a complex trait that attributes to overall expressions of morphological, physiological and biochemical elements, and is also strongly affected by genotype-environment interactions. If the yield is used as the sole breeding criterion for NUE, the breeding process will be slow due to the requirement of multi-year field trials to accurately select elite genotypes [172]. Therefore, the use of secondary selection criteria such as morphological, physiological and biochemical traits is an indirect breeding approach used to develop new varieties for an enhanced NUE [173]. The final grain weight is largely affected by the duration and rate of linear grain growth and is a result of the interplay between potential grain weight (sink) and the actual supply of assimilates per grain during grain filling (source) [174,175]. Thus, the genes playing significant roles in this process should be brought into the research target.

The great number of published GPC related QTLs and their associated markers should be brought into usage in breeding program. Priorities should be given to these QTLs/markers reported in multiple publications (Table 2) and pleiotropic markers for a combinations of desirable traits. Future QTL mapping work should avoid segregations of the known GPC QTLs in the structured mapping populations so that novel genetic factors can be identified.

Phenomics provides a platform to perform non-invasive biological data collection on a large number of plants simultaneously and observations of plant behavior by utilizing new technologies [194]. Since canopy photosynthesis is a major determinant of ultimate yield, and canopy also acts as a reservoir of N and other minerals for recycling into grain [195], high throughput and extensive phenotyping of canopy related traits could boost the identification of high NUE traits. Massive improvements in phenotyping technology in recent years made it possible to characterize those traits with higher accuracy. Non-destructive phenotyping using different sensors and cameras have made significant advances in efficient and reliable phenotyping for NUE studies in wheat [196,197,198]. The high correlations of red and NIR spectra with chlorophyll and N content, biomass, and grain yield have efficiently accelerated the NUE related phenotyping process [198]. Furthermore, modern technologies allow NUE phenotyping by better quantification of biomass, growth rates and transpiration rates (reviewed by Nguyen et al. [173]).

Combining transcriptome and proteome data as well as metabolite profiles to identify co-regulated genes, metabolites and proteins and further superimpose data on known pathways will allow for greater precision and confidence in identifying genes and processes of interest [81]. Finally, the role of proteins in mature grain should be emphasized. Transcriptome studies have shown that more than 30,000 genes are expressed in the developing wheat grain, while proteomic analysis of mature grain has identified only 1125 individual proteins [199]. The wheat storage protein consists of the main components grain protein. It has been reported that manipulating seed storage protein can lead to an improvement in total GPC, thus improving wheat NHI and NUE as a result [200,201]. An in-depth profiling of wheat storage proteins has the potential to improve NUE.

## 10. Conclusions

Enhancing NUE is one of the most effective approaches to achieve high and balanced yield and quality for wheat breeding. To select an appropriate definition of NUE and traits used in the evaluation of different genotypes is essential for wheat NUE related studies. Among the various NUE definitions and related traits, the applications of NHI and GPD will achieve a balanced yield and protein quality. NUE, as a complex quantitative trait, is affected by factors involved in the process of the uptake, assimilation and remobilization of N, including transporter proteins, kinases, key enzymes, circadian regulators, cross-talks between carbon metabolism, TFs and miRNAs, etc. Future genetic improvements in NUE could incorporate multi-omics techniques and take references from the previously reported and verified QTLs and molecular markers to facilitate the QTL-mapping and gene identification process. In addition, exploring other selection criteria related to morphological, physiological and biochemical traits and applying new technologies from phenomics will allow high-throughput phenotyping and accelerate wheat breeding towards a higher NUE.

## Figures and Tables

**Table 1:** Previously published QTLs for GPC.

Cross	Population Type and Size	QTL Detected on Chromosomes	Reference
Ning7840 x Clark	132 RIL	3A, 4B	[102]
Avalon/Hobbit Sib	200 RIL	2B, 6A, 6B, 7A	[103]
DT695/Strongfield	185 DH	1A, 1B, 2A, 2B, 5B, 6B, 7A, 7B	[104]
Gaocheng8901/Zhoumai16	176 RIL	3BL	[105]
Choteau/Yellowstone	97 RIL	3B, 5B	[106]
Berkut/Krichauff	138 DH	1A	[107]
Chara/WW2449	190 DH	4A	[108]
Norstar/winter Manitou, Cappelle Desprez/Norstar, Norstar/Manitou	161 DH for P1, 256 DH for P2, 152 DH for P3	3A, 4A, 5A, 6A, 6B, 7D	[109]
Association mapping	150 cultivars	1A, 2A, 2B, 3A, 3B, 6B	[110]
Forno/Oberkulmer	226 RIL	1B, 3B, 4A, 5A, 5B, 6B, 7A, 7B, 7D	[111]
ITMI	114 RIL	7A, 2D	[112]
Renan x Récital	197 RIL	2A, 3A, 4D, 7D	[113]
PHS132/WL711	100 RIL	1A, 2AS, 3AL, 3BS, 4AS, 4DL, 5BL, 6AL, 7AS, 7DL	[114]
Courtot/Chinese Spring	187 DH	1BL, 6AS	[115]
PHS132/WL711, ITMI population	100 RIL for P1, 110 RIL for P2	1AS, 1BL, 1DL, 2AS, 2AL, 2BL, 2DS, 2DL, 3BS, 4AS, 5BL, 5DL, 6DL, 7AL, 7DS	[116]
AC Karma x 87E03-S2B1	185 DH	4D, 7B	[117]
MG29896/Latino	92 BIL	2AS, 6AS, 7BL	[118]
W7985/Opata85	114 RIL	2A, 2D, 5A, 6D	[119]
Batis/Syn022, Zentos/Syn086	250 BC_2_F_3_ for P1, 150 BC_2_F_3_ for P2	3AL, 4AL, 4BL, 5DL, 7BS, 7DS	[120]
Kukri/Janz	160 DH	1B, 3A, 5A, 5B, 7A	[121]
Neixiang 188/Yanzhan 1	188 RIL	1B, 2A, 2B, 2D, 3A, 3B, 4D, 5B, 5D, 7B, 7D	[122]
MN98550 x MN99394	139 RIL	2A, 5B, 6B, 7A	[123]
Huapei 3/Yumai57	168 DH	3A, 3B, 5D, 6D	[124]
PH82-2/Neixiang 188	240 RIL	3A, 3B, 4A, 5D	[125]
25R26′ and ‘Foster	171 RIL	2A, 2B, 7D	[126]
Association mapping	207 accessions	1B, 2D, 3A, 5D	[127]
UC1113/Kofa	93 RIL	1B, 2A, 2B, 3B, 4A, 5A, 5B, 7A, 7B	[128]
Association mapping	372 accessions	1A, 1B, 1D, 2A, 2B, 2D, 3D, 4B, 5B, 5D, 6A, 6B, 6D, 7B	[129]
Svevo x Ciccio	120 RIL	1A, 2A, 2B, 3B, 4A, 4B, 5A, 6B	[130]
Am3/Laizhou 953	82 IL	1A, 2D, 4B, 5D, 6A, 6B, 6D, 7B	[131]
Xiaoyan 54/Jing 411	182 RIL	4B, 4D, 5A, 6A	[132]
Weimai 8/Luohan 2	302 RIL	2A, 3B, 4A, 5B, 5D, 6B, 7A	[133]
OS9/Q36	164 RIL	6BS, 7DL, 2AS, 5DL, 1AL	[134]
BR34/Grandin	118 RIL	5B	[135]
Weimai 8/Jimai 20, Weimai 8/Yannong 19	485 RIL for P1, 229 RIL for P2	1A, 1B, 2A, 2B, 2D, 3A, 4A, 4B, 4D, 5A, 5B, 5D, 6B, 7A, 7B, 7D	[136]
Louise/Penawawa	188 RIL	3B	[137]
Toisondor/Quebon, CF9107/Quebon, Toisondor/CF9107	91 DH for P1, 90 DH for P2, 140 DH for P3	2A, 2D, 3A, 3B, 5D, 7B, 7D	[40]
RAC875/Kukri	192 DH	2B, 2D, 3A, 4A, 6A, 7A	[138]
CO940610/Platte	185 DH	5B, 6A, 6B, 7B, 7D	[139]
Association mapping	196 accessions	1A, 1B, 1D, 2B, 3B, 4A, 5B, 6A, 6B	[140]
Association mapping	376 cultivars	6A	[141]
Association mapping	214 European varieties	2B, 3A, 3B, 5A, 5B, 6D, 7B	[142]
Association mapping	118 accessions	3B, 5B	[143]
Drysdale/Gladius	155 RIL	2B, 2D, 3B, 5A	[144]
WCB414/WCB617	163 RIL	1A, 1B, 2B, 2D, 3D, 4B, 5B, 6B, 7B	[145]
Huapei 3/Yumai57, Nuomai 1/Gaocheng 8901, Shannong 01–35/Gaocheng 9411	168 DH for P1, 256 RIL for P2, 182 RIL for P3	1B, 1D, 2A, 2B, 2D, 3B, 4B, 5B, 6D, 7A	[146]
Association mapping	407 varieties	5A, 5B, 6D	[147]
Yumechikara/Kitahonami	94 DH	2B	[148]
Ning7840/Clark	132 RIL	3A, 4BS, 5AL, 5BL	[149]
Avonlea/Duilio	RIL	1B, 2B, 3B, 4A, 5A, 7A, 7B	[37]
WH542/Synthetic wheat	286 RIL	2A, 3A	[150]
Association mapping	299 varieties	1D, 2B, 2D, 4B	[15]
WL711/C306	206 RIL	1B, 1D, 3B, 3D, 5D, 7A	[151]
NAM population	2038 RIL	1A, 1B, 4A, 4B, 4D, 5A, 6A, 6D, 7B, 7D	[152]
TN18/LM6	184 RIL	1B, 4B, 6A	[153]
Association mapping	96 accessions	1B, 2B, 3A, 3D, 4A, 7B	[154]
NAM population	175 RIL	1A, 1B, 2A, 2B, 3A, 3B, 4A, 4D, 5B, 7B, 7D	[155]
Cutler/AC Barrie, Attila/CDC Go, Peace/Carberry, Peace/CDC Stanley	698 RIL in total	2D, 4B, 5A	[156]

Note: DH: double haploid; RIL: recombinant inbreed line; IL: introgression line; P1, P2 and P3 represent population 1, population 2 and population 3, respectively.

**Table 2:** Repeatedly published markers linked with GPC QTL.

Marker	Chr.	Trait	References
wPt1167	1A	GPC	[40,129]
wPt7094, wPt8267, wPt0328	1B	GPC	[40,129]
barc81, barc188, Xgwm153, NP251	1B	GPC, TKW	[121,176]
gwm403, bcd442	1B	GPC, TKW	[116]
Xwmc419, E35M4714	1B	GPC, TKW	[127]
Bx7-NW2242	1B	GPC	[121]
gwm413, cfd65	1B	GPC	[151]
GENE-0129_123, Excalibur_c63563_370, RAC875_rep_c111494_195, BS00063551_51 1B	1B	GPC	[154]
D-1190331, S-3222160	1B	GPC	[153]
cfd61, cfd72	1D	GPC	[151]
Xwmc455, Xgwm515, wms473	2A	GPC, TKW	[121,177]
XksuD18, Xgwm400, Xgwm636, Xgwm614	2A	GPC, TKW	[113,178]
Xbarc15, Xgwm558, Xgwm294, gwm614, bcd1184, bcd152, bcd543	2A	GPC, TKW	[116,131,179]
Xwmc630b, Xwmc453, TC82001, Xgwm372c	2A	GPC	[130]
1267600, 1138191	2A	GPC	[180]
Xwmc245, Xgwm271, Xgpw4382, Xgpw3215, barc0013	2B	GPC, TKW	[123,148,181]
Xgwm1249, wPt-1294, gwm319	2B	GPC	[116]
Xwmc344.5, Xwmc344.1	2B	GPC, TKW	[131,182]
Xgwm410, Xbarc183, Xgwm644, Xbrac55, Xbarc7	2B	GPC, TKW	[103,183]
Ppd-B1, wPt-3561	2B	GPC	[144]
FdGOGAT-B	2B	GPC	[140]
RFL_Contig1445_1192	2B	GPC	[154]
1083804, 1117983, 2303802, 2275590	2B	GPC	[180]
Ppd-D1, gpw332	2D	GPC	[140,144]
wmc18	2D	GPC, TKW	[127,184]
wPt-8330–wPt-7901	2D	GPC, TKW	[129,177]
wms210, wmc111, wPt6657	2D	GPC, TKW	[121,142]
gwm261, cdo1379, bcd262	2D	GPC, TKW	[116,185]
Xbarc86, Xwmc21, Xswes107	3A	GPC, TKW	[124,177]
Excalibur_c48047_90, RAC875_c28721_290	3A	GPC	[154]
D_521287, Xgwm389	3B	GPC, TKW	[130]
wPt-7961, wPt-9066	3B	GPC, TKW	[144,186]
Xwmc3, Xwmc56, Xbarc68.1, wmc418	3B	GPC	[121,125,133]
barc147, gwm493	3B	GPC	[128,129]
BS00026471_51	3B	GPC	[154]
cfb3059, cfb3375	3B	GPC	[151]
Kukri_c7658_229	3D	GPC	[154]
wmc443, gpw4136	3D	GPC	[151]
dupw4, barc170	4A	GPC	[128,140]
Xwmc516, BE517017	4A	GPC, TKW	[133]
Xgwm0160, Xgwm0832, DuPw202	4A	GPC, TKW	[125,187]
Tdurum_contig100702_265	4A	GPC	[154]
3942314, 5323574	4A	GPC	[180]
D_310555, Xgwm251	4B	GPC, TKW	[130]
Xgwm368, Xwmc617, IWA4662, Rht-B1	4B	GPC, TKW	[102,188]
IWA482, IWA1846, IWA4662	4B	GPC, TKW	[149,189]
D-1380792, D-1094306	4B	GPC	[153]
Xcfd193, Xcfd71, wmc457	4D	GPC, TKW	[113,117,121]
Xwmc52	4D	GPC, TKW	[117]
Xwmc331, Xgwm194, XwPt9094	4D	GPC, TKW	[121,123]
wsnp_Ex_rep_c107564_91144523	4D	GPC	[154]
Xbarc330, XwPt9094	5A	GPC, TKW	[123,185]
Xbarc180, Xbarc141, Xgwm154	5A	GPC, TKW	[102,132]
Xgwm540, Xgwm499, NW2071, BE495277_339	5B	GPC	[106,121,128,139]
wPt3503, Xcfe186, Xgwm639	5B	GPC, TKW	[146,189]
Xbarc110, Xgwm544.1	5B	GPC, TKW	[117,133]
Xbarc234.1, Xfcp273, XwPt9006	5B	GPC, TKW	[123,133,135]
Xissr854.1, Xwmc73	5B	GPC, TKW	[104,190]
Xwmc215, Xcfd29, vrnD, Xbarc320, Xcfe242.2	5D	GPC, TKW	[121,124,131,133,185]
Xcfd18-Ha	5D	GPC, TKW	[125,191]
wPt-8030, gwm174	5D	GPC, TKW	[133,140]
barc130, gwm190	5D	GPC	[151]
Xcfe273.2, Xcfe273.1	6A	GPC, TKW	[129,190]
Xcfd80.2, Xbarc1055, Xwmc553, Xwmc807, Xbarc37	6A	GPC, TKW	[102,132,141]
D-1112857, S-2362461	6A	GPC	[153]
Xbarc146, Xgwm88, Xswes131.2	6B	GPC, TKW	[131,190]
Xgwm133, Xbarc24, Xcfd190, Xgwm219	6B	GPC, TKW	[103,141]
Xcfd42–Xcfd13	6D	GPC	[124,146]
Xfba85, Xgwm469	6D	GPC, TKW	[119,185]
Xgwm573, Xwmc9, Xbarc108, gwm1171, gwm276, ksuH9	7A	GPC, TKW	[103,104,116,121,178,187]
wmc017, NW1257, Xcfa2174.1	7A	GPC, TKW	[121,133,188]
wmc168, barc219	7A	GPC, TKW	[128,133]
Xgwm473, Xedm16.1	7A	GPC, TKW	[131]
wmc525, cwem53b	7A	GPC	[151]
Xbarc65, Xcfe75	7B	GPC, TKW	[131]
Xgwm569, Xbarc278, Xbarc1181, Xwmc276, Xgwm146, barc50, wmc311	7B	GPC, TKW	[141]
Vrn-D3, wPt-3727, Xgwm437, Xbarc128, Xcfd46, Xgwm295	7D	GPC, TKW	[139]
BobWhite_c19429_95	7D	GPC	[154]
Xbarc0176, Xcfd69	7D	GPC, TKW	[192]
Xcfd4, Xgwm44	7D	GPC, TKW	[131]
Xgwm111	7D	GPC, TKW	[126,193]

## Data Availability

The data presented in this study are available in the article.
